# Diaphragm Dysfunction: Diagnostic Approaches and Management Strategies

**DOI:** 10.3390/jcm5120113

**Published:** 2016-12-05

**Authors:** Bruno-Pierre Dubé, Martin Dres

**Affiliations:** 1 Département de Médecine, Service de Pneumologie, Hôpital Hôtel-Dieu du Centre Hospitalier de I’Université de Montréal (CHUM), Montréal, QC H2W 1T8, Canada; 2 AP-HP, Groupe Hospitalier Pitié-Salpêtrière Charles Foix, Service de Pneumologie et Réanimation Médicale (Département “R3S”), F-75013 Paris, France; martin.dres@aphp.fr

**Keywords:** diaphragm, diaphragm dysfunction, phrenic nerve stimulation, diaphragm ultrasound

## Abstract

The diaphragm is the main inspiratory muscle, and its dysfunction can lead to significant adverse clinical consequences. The aim of this review is to provide clinicians with an overview of the main causes of uni- and bi-lateral diaphragm dysfunction, explore the clinical and physiological consequences of the disease on lung function, exercise physiology and sleep and review the available diagnostic tools used in the evaluation of diaphragm function. A particular emphasis is placed on the clinical significance of diaphragm weakness in the intensive care unit setting and the use of ultrasound to evaluate diaphragmatic action.

## 1. Introduction

The diaphragm is the main respiratory muscle. Its dysfunction can be associated with the presence of respiratory symptoms, exercise intolerance, sleep disturbances and, in the more severe cases, have a negative impact on survival. Uni- and bilateral diaphragm dysfunction diagnosis and management may be problematic for the clinician because of its relative rarity, its sometimes subtle clinical manifestations and because of difficulties in obtaining a physiologically-confirmed diagnosis. As such, diaphragm dysfunction is probably underdiagnosed, but should not be neglected, as it can negatively impact quality of life, can be a marker of disease severity and, in some instances, such as in the intensive care unit, be a prognostic marker. The aim of this review is in part to provide clinicians with an overview of the possible causes of diaphragm dysfunction, but also to explore the diagnostic methods available to investigate diaphragm function and to review current and future therapeutic strategies available to patients with diaphragm weakness.

## 2. Anatomical Considerations

The diaphragm is the musculo-fibrous membrane that separates the thoracic and abdominal cavities. It is constituted of a non-contractile central fibrous portion and a peripheral muscular section that is partitioned into the sternal, costal and lumbar muscular groups. The muscular component of the diaphragm displays approximately equal proportions of slow, fatigue-resistant (Type 1) and fast (Type II) fibers, a finding that reflects its roles as an actor in both the low-intensity, perpetual cycle of breathing and in more rapid and strenuous settings, such as talking, singing, sneezing, defecation and in situations of acutely-increased ventilation [1,2,3]. The zone of apposition (ZOA) is the area on the lateral sides of the lower thorax where the muscular diaphragmatic fibers run parallel and in close apposition to the chest wall. 

Afferent neurological inputs to the diaphragm originate almost exclusively from the phrenic nerves, which stem from the third, fourth and fifth cervical nerves bilaterally. At the level of the neck, both phrenic nerves descend anteriorly to the scalene muscles and enter the thorax between the subclavian arteries and veins. The right phrenic nerve runs caudally anterior to the brachiocephalic trunk, borders the right atrium and enters the abdominal cavity through the caval hiatus. The left phrenic nerve runs caudally along the left ventricle and enters the diaphragm on its own. From the abdominal side of the diaphragm, the phrenic nerves divide in four branches that will allow innervation of the entire muscle [4,5].

The thickness of the diaphragm is variable over its surface, with tapering from the anterior to posterior costal regions and from its costal insertions to the central tendon. During contractile shortening, the shape of the diaphragm changes little, and most of the shortening is translated into axial descent. Under normal conditions, the diaphragm acts like a piston within the chest, generating flow as its dome descends within the thoracic cavity, while it displaces the abdominal contents caudally and elevates the lower thorax. The negative intrathoracic pressure created by this action causes an inflow of air from the mouth to the lung, generating tidal volume.

## 3. Etiology of Diaphragmatic Dysfunction

### 3.1. Confirming the Diagnosis

Unilateral diaphragm paralysis is often first suspected after the finding of an abnormally elevated hemidiaphragm on a chest radiograph, which can be defined as a right hemidiaphragm sitting >2 cm higher than its left counterpart or a left hemidiaphragm sitting equal or higher than the right hemidiaphragm. This finding is relatively common, but should not be taken as a decisive indication of diaphragmatic paralysis, as a chest radiograph has high sensitivity (90%), but unacceptably low false positive findings (positive predictive value of 33%) for the diagnosis of diaphragm dysfunction [6]. In accordance, the first step in the evaluation of an elevated hemidiaphragm on a chest radiograph should be the evaluation of possible alternative explanations for this finding (Table 1). Among these, congenital diaphragmatic hernias, atelectasis of various causes, pulmonary and diaphragmatic masses are usually easily identifiable on a simple chest radiograph, whereas intra-abdominal processes, such as ascites of subphrenic masses or abscesses, may require additional imaging. In the presence of a “true” diaphragmatic elevation, the chest radiograph will show a homogenous, regular and continuous hemidiaphragm that is abnormally elevated, with no ipsilateral retraction.

### 3.2. Unilateral Diaphragm Weakness (Table 2)

Traumatic lesions are one of the most common causes of “true” unilateral diaphragm paralysis. In particular, coronary artery bypass grafting (CABG) surgery is frequently associated with lesions of the phrenic nerves resulting in post-operative diaphragmatic paralysis, with incidences reported as varying from 1% to 60% [7,8,9,10,11]. One of the reasons for this wide estimate probably lies in the various surgical techniques used during CABG: harvesting of the internal mammary artery (IMA) and the use of topical ice slush for cardiac cooling are associated with an increased risk of phrenic injury [7,9,10,11,12,13,14], the former because of the close anatomical relationship of the nerve and the IMA and the latter because of the traumatic demyelinating injury to the phrenic nerved induced by the cold temperature. 

The clinical consequences of phrenic injury following CABG seem dependent on the presence of pre-existing respiratory diseases, such as chronic pulmonary obstructive disease (COPD). In a study exploring the short and midterm prognosis of patients following CABG, the presence of phrenic nerve injury was associated with worse outcome regarding the length of hospitalization, intensive care unit (ICU) stay, need for reintubation, post-operative pneumonia and bronchospasm, but only in patients with concurrent COPD [8]. Although the precise explanation for this phenomenon remains unclear, it is possible that the negative clinical outcomes observed in this group of patients represent the additive effects of diaphragm weakness on an already compromised lung function. Whether patients with other underlying lung anomalies such as interstitial lung disease would also be more prone to adverse clinical outcomes in this setting is unknown. In this study, patients with COPD also had decreased survival and midterm quality of life, a finding that, again, possibly reflects the negative impact of an additional injury to the already decreased pulmonary reserve of these patients. 

Post-operative unilateral diaphragm palsy has also been described after neck surgery [15,16,17,18] and lung or liver transplantation [19,20,21,22], where it has been associated with short-term respiratory complications. 

More rarely, inflammatory neurological conditions, such as neuralgic amyotrophy (Parsonage–Turner syndrome) present with unilateral phrenic palsy, which usually resolves over one to three years [23,24,25,26,27]. 

In many cases, isolated phrenic neuropathy with no apparent cause will be categorized as idiopathic. Large-scale, quality studies investigating the pathogenesis and potential therapeutic interventions for these cases are currently lacking, owing in part to the rarity of the disease and the heterogeneous nature of their clinical presentation. Infectious processes have been proposed as the underlying cause of so-called unilateral phrenic palsies in light of the partial therapeutic success observed with the treatment of patients presenting with acute idiopathic phrenic neuropathy with intravenous immunoglobulins [28] and valacyclovir [29]. These findings must however be interpreted with caution, as they rely on small case series and often do not include control groups. Table 2 summarizes the main causes of unilateral diaphragm dysfunction.

### 3.3. Bilateral Diaphragm Weakness

In contrast to unilateral disease, bilateral diaphragmatic weakness is most often encountered in the setting of a generalized degenerative muscular or neurological disorder (Table 3).

In motor neuron disease, the severity of diaphragm involvement usually mirrors that of the peripheral muscle, although some cases present with respiratory failure as an initial manifestation [30,31,32]. The clinical importance of respiratory muscle weakness in these neuromuscular disorders cannot be overstated and should be rigorously and repeatedly investigated in all patients, as the presence of diaphragm weakness is a negative prognostic marker in many of these pathologies, such as amyotrophic lateral sclerosis, Guillain–Barré syndrome and the hereditary myopathies (see Section 5) [33,34,35,36,37].

Shrinking lung syndrome is a rare, but potentially serious complication of systemic lupus erythematosus (SLE), which is characterized by a progressive decline in lung volumes without concomitant interstitial lung disease or pleural disease. Although is it generally thought to be a consequence of an inflammatory process targeting the diaphragm [38] or the phrenic nerves [39], one study reported normal or near-normal diaphragm contractility in nine out of 12 patients with the syndrome, assessed using phrenic nerve stimulation [40]. It has been suggested that the restrictive physiological defect found in these patients may be more dependent on an inflammatory pleural process impairing normal ventilation [41], possibly coupled to a reflex concomitant inhibition of diaphragm activation [42]. Among other collagen-tissue diseases, dermatopolymyositis is also frequently associated with bilateral diaphragm weakness and may be underdiagnosed [43].

More rarely, disease of the neuromuscular junction [44], hypothyroidism [45,46] and malnutrition [47] can also present with bilateral diaphragm weakness, the latter being reversible with renutrition. 

Diseases presenting with chronic lung hyperinflation such as COPD are another frequent cause of abnormal diaphragm contractility. In these patients, the pressure-generating capacity of the diaphragm is reduced [48,49,50], mainly due to lung hyperinflation, which shortens its muscle fibers and places the diaphragm at a mechanical disadvantage by having it contract on the steep part of its length-tension curve. Of note, when their diaphragm contractility is assessed after correction for the severity of the underlying hyperinflation, COPD patients show normal or even supra-normal contractility compared with control subjects [48]. 

Finally, in the ICU setting, acquired diaphragm dysfunction has recently grown to become a topic of significant interest because of its negative clinical impact on weaning outcome, length of mechanical ventilation (MV), survival and long-term outcome [51,52,53,54,55,56,57,58,59,60,61,62,63]. One of the main contributors to diaphragm dysfunction in this context is mechanical ventilation, which has been shown to induce significant diaphragm muscle fiber atrophy that can be detected within the first 24 h of MV in humans and even earlier in animal models [64,65,66]. The molecular and cellular mechanisms implicated in this phenomenon include an increased oxidative stress load on the diaphragm [64,65,67,68], downregulation of protein synthesis [64,69] and activation of proteolytic pathways [68,69,70,71]. Although a causal relationship between MV-induced diaphragm atrophy and loss of function has not been formally demonstrated, there is considerable evidence that continuous MV induces a measurable and significant diaphragm weakness [71,72,73,74,75,76,77], and this phenomenon may be exacerbated by the use of neuromuscular blocking agents [78]. In addition, there is also evidence that diaphragm dysfunction is already present in a large proportion of critically ill patients at the time of admission to the ICU [52,72], presumably before the onset of the negative effects of MV, suggesting that other factors may play a role in the development of ICU-acquired diaphragm dysfunction. Among these, the presence of sepsis has been associated with prominent diaphragm weakness [52,79,80,81,82,83], possibly via similar mechanisms that are observed in sepsis-related myopathy [84]. In addition, the occurrence of diaphragm dysfunction is poorly related to the presence of ICU-acquired peripheral muscle weakness, suggesting that they share different determinants [53]. Strategies to offset the development of diaphragm disease in the ICU are currently under investigation (see Section 6). 

## 4. Clinical Presentation and Diagnosis

Unilateral diaphragm weakness can be seemingly asymptomatic. In the presence of more severe diaphragm paresis or in patients with underlying obesity or cardiorespiratory diseases, orthopnea, dyspnea when bending forward, coughing, chest pain and dyspnea on exertion may become evident [85,86,87,88], as can symptoms of sleep-disordered breathing [88,89]. In contrast, patients presenting with bilateral diaphragm weakness very frequently report severe dyspnea when supine and during exertion [87,90,91,92,93] and are likely to show features of sleep-disordered breathing [37,94,95,96,97]. In all cases, a careful evaluation is required to try to identify the etiology of diaphragm weakness, and clinical testing is required to confirm the presence and the severity of the disease.

### 4.1. Maximal Inspiratory Pressures and Phrenic Nerve Stimulation

The measurement of inspiratory pressure at the mouth during a maximal inspiratory effort against a closed mouthpiece (maximal inspiratory pressure (MIP)) is widely used as a test of respiratory muscle function [98]. It has the advantages of being simple to perform and well tolerated. However, this measurement is effort dependent [99,100], represents the combined action of all inspiratory muscles rather than isolated diaphragmatic contraction and is associated with widely-variable predicted values [100,101,102,103,104] that limit its use as a tool to evaluate and follow diaphragmatic contractility. A MIP < −80 cmH_2_O is generally thought to exclude clinically-significant inspiratory muscle weakness [98], and unilateral and bilateral diaphragm paralysis can be expected to decrease MIP in the ranges of 60% [105] and <30% [90] of the predicted values, respectively. However, these values may be greatly impacted by the presence of underlying obstructive or restrictive lung disease [86].

The gold standard method of quantifying the mechanical function of the diaphragm is the measurement of the negative pressure generated by diaphragm contraction in response to phrenic nerve stimulation [98]. During the stimulation, this pressure can be monitored using either the difference between oesophageal and gastric pressures (twitch transdiaphragmatic pressure, Pdi,tw), or directly at the mouth or, in intubated patients, at the end of the endotracheal tube (twitch mouth or tracheal pressures, Pmo,tw and Ptr,tw) [106,107,108,109]. Although these variables can be used to quantify diaphragm contractility during a sniff manoeuver (Pdi,sn) or during maximal inspiratory efforts against a closed airway (Pdi,max), these maneuvers are heavily dependent on patient effort and are therefore prone to variation. On the other hand, stimulation of the phrenic nerves produces a non-volitional contraction of the diaphragm. Transcutaneous electrical phrenic stimulation can be performed at the level of the neck uni- or bi-laterally, but has the disadvantages of being uncomfortable for some patients and technically more difficult in patients with obesity or anatomical variations. Magnetic stimulation of the phrenic nerves is painless, can be applied bilaterally at the level of the cervical spine [110] or uni- or bi-laterally at the neck, is reproducible and easy to perform. A Pdi,tw < 15 cmH_2_O and a Pmo,tw or Ptr,tw < 11 cmH_2_O [52,108] are generally indicative of diaphragm dysfunction. Overall, phrenic stimulation techniques require considerable expertise, specialized equipment, are time consuming and, as such, are poorly adapted to routine clinical use. Other means of evaluating diaphragm function are therefore often used.

### 4.2. Lung Function Testing

Lung function testing is frequently performed as a first-line test to assess and quantify the physiological impact of diaphragm weakness. Unilateral diaphragm weakness is usually associated with a mild decrease in vital capacity (VC), to approximately 75% of the predicted value [86,105], with a further 10% to 20% decrease in the supine position [105], while functional residual capacity (FRC) and total lung capacity (TLC) are usually preserved [86,105]. In bilateral diaphragm weakness, VC usually reaches mean values of approximately 50% predicted and can further decrease by 30% to 50% when supine [90]. TLC can also be reduced, while residual volume (RV) can be elevated [111]. Of note, the magnitude of the fall in VC in the supine position has been shown to be correlated to Pdi,sn in this population [111].

### 4.3. Fluoroscopy

Dynamic evaluation of diaphragmatic movement using fluoroscopy has long been used to evaluate possible unilateral diaphragmatic weakness [112]. During a sniff manoeuver, a paradoxical upward motion of the abnormal hemidiaphragm may be observed, which confirms the diagnosis. Despite its ease of use, the test has several drawbacks: it has only rarely been compared to a reference technique, making estimates of its sensitivity and specificity imprecise; it is dependent on patient effort and cooperation and only provides a semi-quantitative evaluation of diaphragm function, making repetitive prospective measurements difficult to compare. In addition, it should not be used in the setting of bilateral diaphragm weakness, as the abnormal breathing pattern observed in this setting may obscure radiological findings and result in false negatives [98,113,114,115]. 

### 4.4. Ultrasonography

Ultrasound evaluation of the diaphragm is simple, non-invasive, readily available at the bedside and increasingly used both in the clinical and research settings [116]. The main variables that can be assessed using this technique include the static measurement of diaphragm thickness (Tdi) and the more dynamic evaluation of inspiratory diaphragm thickening fraction (TFdi) and excursion (EXdi). 

Measurement of Tdi and TFdi are usually performed using a high-frequency linear array transducer positioned at the level of the ZOA, where the diaphragm is identified as a three-layered structure comprising two hyperechoic lines representing the pleural and peritoneal membranes and a middle hypoechoic layer representing the diaphragmatic muscle itself [117]. Tdi is measured at end-expiration, while TFdi requires the measurement of both end-expiratory and end-inspiratory diaphragm thicknesses and is computed as [(inspiratory thickness − expiratory thickness)/end-expiratory thickness], expressed as a percentage. Tdi measurements are reproducible [118,119,120] and correlated to direct anatomical measurements [121]. The lower limit of normal for Tdi has been reported to be 0.15 cm in healthy subjects [120] and patients with COPD [122], but it is unclear whether a Tdi value below this threshold is representative of diaphragm dysfunction. Indeed, static diaphragm thickness can vary with posture [123] and stature [124], and in reports of patients with diaphragm paresis, the majority of the subjects had Tdi > 0.15 cm [125,126,127]. However, the temporal change in Tdi was correlated to the change in VC in patients with spontaneous recovery of diaphragm function [125]. In the ICU setting, Tdi in itself is a poor predictor of weaning outcome [58,59].

In contrast, TFdi has been shown to be correlated to the pressure-generating capacity of the diaphragm [58,128], to the work of breathing and respiratory effort [118,129] and can be used as a valid tool to identify diaphragm dysfunction [130,131], monitor its temporal changes [29,125] and may predict weaning outcomes in patients under invasive or non-invasive ventilation [53,58,59,132,133]. The reported lower limit of the normal value for TFdi is 20% in healthy subjects and patients with COPD [120,122]. 

EXdi is usually measured using a curvilinear probe positioned in the infra-hepatic region [117], and has similarly been shown to be reproducible [134], related to transdiaphragmatic pressure [135] and to weaning outcome in intubated patients [57]. The lower limit of normal of EXdi is 3.7 cm in women and 4.7 cm in men, during maximal inspiratory effort [134].

These findings, coupled to the observation that ultrasound evaluation of the diaphragm has been shown to be more sensitive than fluoroscopy for the evaluation of diaphragm weakness [136], position ultrasound at the foreground of the clinically-available tools to quantify and follow diaphragm function.

## 5. Complementary Investigations

### 5.1. Sleep Studies

Sleep-disordered breathing (SDB) may be more prevalent than previously thought in patients with unilateral diaphragm weakness [88,89]. A recent study [88] showed increased neural respiratory drive to the diaphragm during non-rapid eye movement (NREM) sleep and a significantly higher respiratory disturbance index (RDI) in this population compared with control subjects, even in the absence of diurnal symptoms. Most respiratory events seem to be central hypopnea secondary to weakness of the respiratory pump, even in the setting of compensatory increased neural drive to breathe, and were particularly prevalent in patients with more severe diaphragm weakness (unilateral Pdi,tw < 5 cmH_2_O) [88]. In accordance, polysomnography could be considered in this population even in the absence of diurnal symptoms [89].

In patients with bilateral diaphragm disease, SDB and hypoventilation are much more common, especially as the disease progresses or when there is concomitant involvement of the accessory respiratory and pharyngeal musculature [97]. A simple five-item questionnaire has recently been shown to be a valuable tool in the prediction of SBD in patients with diaphragm paralysis [137]. At the clinical level, however, age, lung function tests results and daytime symptoms have been reported to be poor predictors of the presence of SDB in this population [95], and as such, polysomnography should be considered early in the evaluation of these patients to allow prompt treatment. In fact, polysomnography testing with concomitant non-invasive ventilation titration could be routinely considered in these patients whenever possible, as this approach allows both a diagnostic and therapeutic evaluation (see Section 6) [97,138].

### 5.2. Cardiopulmonary Exercise Testing

Although few studies have evaluated the value of CPET in patients with diaphragm weakness, the evaluation of exercise performance in this population may help to demonstrate negative repercussions of the disease in patients that are asymptomatic at rest, quantify the level of respiratory impairment and monitor clinical evolution or improvement (spontaneous or following a therapeutic intervention). 

During exercise, patients with unilateral diaphragm paralysis show a slightly decreased exercise endurance time, peak minute ventilation and higher oxygen cost of breathing than control subjects, and these anomalies were even greater in patients with bilateral weakness [87]. Although mean peak oxygen uptake (VO_2_) is slightly decreased in patients with bilateral diaphragm weakness [87,90], it can be preserved in those with unilateral disease despite the aforementioned anomalies, especially if respiratory muscle endurance is preserved and compensatory mechanisms have developed [87], highlighting the need for a meticulous interpretation of CPET results in these patients.

## 6. Prognosis

The prognostic and clinical evolutions of uni- and bi-lateral diaphragm weakness are highly variable and related to its underlying etiology. Patients with unilateral disease have excellent vital prognosis, but the degree of spontaneous recovery is variable and seems more frequent in patients with post-operative phrenic injury [13,14,15,16] than in those with idiopathic disease [139,140]. In patients with neuralgic amyotrophy or idiopathic disease, partial recovery of diaphragm function can be expected, but sometimes requires years [29,125,141].

Bilateral diaphragm weakness secondary to neuralgic amyotrophy can also be expected to improve after several years of follow-up [93,125], as can cases labeled as idiopathic. However, in the context of degenerative myopathies or neurological diseases, respiratory muscle weakness frequently progresses relentlessly with the underlying disease and will very frequently negatively impact quality of life and vital prognosis [31,34,35,36,142]. Although acute high spinal cord injury will frequently cause acute respiratory failure requiring ventilatory support, spontaneous diaphragm motor recovery can be observed within the first month, presumably because of the resorption of local edema, or even up to 14 months after the initial injury [143].

In the ICU setting, diaphragm dysfunction is associated with adverse prognosis whether it is diagnosed at admission (increased length of stay and mortality) or at the time of weaning from mechanical ventilation (increased weaning failure) [51,52,53,54,55,56,57,58,59,60,61,62,63].

## 7. Therapeutic Management

Treatment of diaphragm weakness should first begin with the optimal medical management of concurrent conditions, such as obesity, deconditioning and COPD, as the repercussions of respiratory muscle failure will be further amplified by these conditions. Inspiratory muscle training (IMT) using either resistive or threshold loading may result in increased maximal inspiratory pressures in patients with COPD and may contribute to improvements in exercise performance and dyspnea [144,145]. In patients with spinal cord injury or under mechanical ventilation, IMT often results in increased inspiratory muscle strength, although further research is required to evaluate whether this translates into systematic improvements of clinical endpoints [146,147,148]. Patients with phrenic nerve damage following CABG may also benefit from IMT, which can lead to a higher chance of partial or complete recovery [149]. In addition, when sleep-disordered breathing is present (whether as a consequence of diaphragm weakness or as a concomitant disease), continuous positive airway pressure (CPAP) or, in the setting of more severe diaphragm weakness and/or nocturnal hypoventilation, non-invasive bi-level airway pressure (BPAP) are the treatments of choice.

### 7.1. Unilateral Diaphragm Weakness

Asymptomatic patients with unilateral diaphragm weakness generally do not require treatment. In the case of patients with concomitant chronic respiratory or cardiac disease, transient ventilatory support may be required in situations of cardiac or respiratory instability, such as with respiratory infections, pulmonary edema or bronchospasm.

If, after a reasonable observation period (≥12 months), repeated testing shows persistent diaphragm weakness and patients describe resting or exertional dyspnea, diaphragmatic surgical plication should be considered. During this procedure, the weak hemidiaphragm is immobilized with surgically-created folds, reducing its paradoxical movement during breathing. In turn, this will decrease the workload and susceptibility to fatigue of the contralateral hemidiaphragm and improve ventilation/perfusion anomalies on the ipsilateral lung base. In carefully-selected patients, the surgery may lead to significant improvement of resting lung function and exercise capacity, but, more importantly, to impressive gains in quality of life [150]. In a case-series of 41 patients that underwent diaphragm plication, forced vital capacity (FVC), forced expiratory volume in 1 s (FEV_1_), FRC and TLC improved by approximately 20% at six months, and these benefits were sustained at 48 months after surgery. Dyspnea (graded on the medical research council scale) decreased by two units, and this improvement was also sustained at 48 months. In another report that included 25 patients, quality of life (measured using St. George’s Respiratory Questionnaire) improved by almost 30 points one year after surgery, a change that is largely above the minimally-important clinical difference of four points [151]. Of note, morbid obesity is a contra-indication to surgical plication and should be addressed before considering the procedure [150]. In some cases of acute unilateral diaphragm paralysis thought to be secondary to viral infection, rapid resolution of the weakness was observed after treatment with valacyclovir [29]. Further studies are required to better understand and delineate the role of antiviral therapy in this setting. Diaphragmatic pacing is not a therapeutic option for unilateral disease.

### 7.2. Bilateral Diaphragm Weakness

Treatment of bilateral diaphragm weakness should first be directed at its underlying cause, when possible. For example, bilateral disease due to shrinking lung syndrome [42,152,153,154], connective-tissue disease [43], hypothyroidism [45,46] or malnutrition [47] may improve with adequate treatment of the underlying disease. Some evidence suggests that bilateral diaphragm weakness may improve with inspiratory muscle training, but further studies on this subject are needed [155,156].

In many patients with degenerative neurological or muscular diseases, diaphragm weakness slowly progresses to chronic respiratory failure. The mainstay of treatment in this setting is non-invasive positive pressure ventilation (NPPV). Criteria used to initiate NPPV in these patients may vary according to the underlying diagnosis and clinical evaluation, but it is generally recognized that diurnal hypercapnia (PaCO_2_ > 45 mmHg), significant nocturnal hypoventilation (oxygen saturation <88% for >5 consecutive minutes), MIP < 60% predicted, FVC < 50% predicted or VC < 20 mL/kg should all prompt NPPV initiation [157,158,159,160,161]. Of note, NPPV institution should not be delayed in these patients, as it can significantly improve the rate of lung function deterioration, quality of life and survival, especially in patients with amyotrophic lateral sclerosis [161,162,163,164,165,166,167,168,169].

In highly selected ventilator-dependent patients with bilateral diaphragm weakness, diaphragm pacing may be indicated. A complete review of the technical aspects of this technique is beyond the scope of this text, and recent high-quality reviews have summarized the available knowledge on this subject [170]. Briefly, the phrenic nerves can be stimulated using implanted electrodes inserted either directly at the thoracic level or on the abdominal aspect of the diaphragm. The main goal of phrenic nerve pacing in these patients is the recovery of a more “physiological” and effective diaphragmatic contraction that can lead to a reduction or removal of ventilatory support. In order to be beneficial, phrenic nerve pacing must be performed in candidates meeting the following criteria: clearly demonstrated bilateral diaphragm weakness (ideally evaluated using Pdi,tw) and ventilatory failure requiring ventilatory support and intact phrenic nerve function (as demonstrated by normal electrical conduction latencies after phrenic nerve stimulation). In addition, adequate cognitive status and the absence of severe concomitant lung or chest wall diseases are required. The ideal candidates for phrenic nerve pacing are patients with upper level spinal cord injury that are still ventilator dependent at least three months after the initial injury: the beneficial effects of pacing were mostly demonstrated in this population, with the majority of patients being liberated from ventilatory support [171,172,173]. Successful cases have also been reported with patients with congenital hypoventilation [174,175,176] syndrome and other neurological diseases [177]. Some data originally suggested a possible benefit of phrenic nerve pacing in patients with ALS [178,179], but two recent, prospective randomized trials evaluating the effect of this intervention in this setting had to be prematurely stopped because of a decrease in survival in the treatment arms [180,181]. In all cases, evaluation for phrenic pacing should be performed in specialized centers with experience in the technique. 

In the ICU setting, strategies aiming at preventing or reversing diaphragm dysfunction are currently being investigated. Among these, the preferential use of partially supported ventilatory modes, rather than controlled ventilation, may prevent diaphragm weakness by promoting its continuous activation [182,183]. Similarly, the use of temporary phrenic stimulation shows promise as a tool to mitigate the negative effects of MV, but remains to be explored in future studies [184,185,186,187].

**Figure 1:**
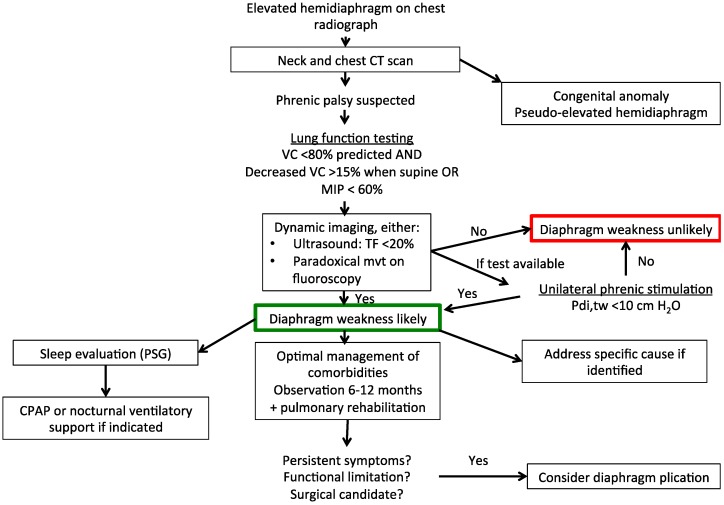
Suggested diagnostic and therapeutic algorithm for unilateral diaphragm weakness. CT, computed tomography; VC, vital capacity; MIP, maximal inspiratory pressure; TF, thickening fraction of the diaphragm; PSG, polysomnography; CPAP, continuous positive airway pressure; Pdi,tw, twitch transdiaphragmatic pressure.

**Figure 2:**
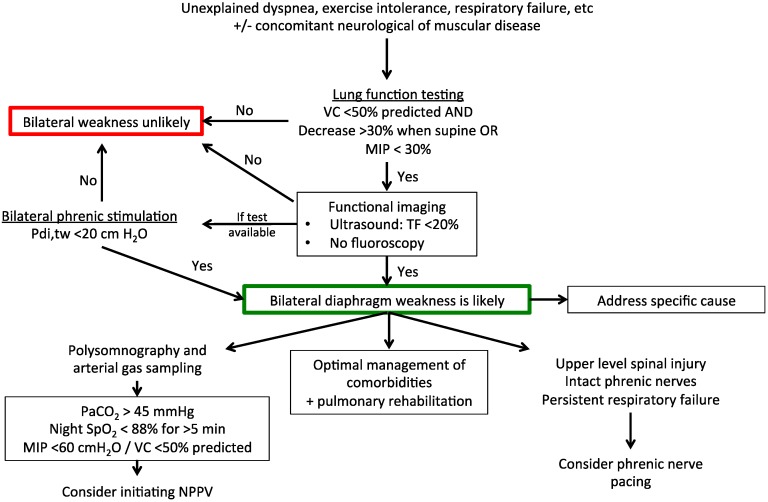
Suggested diagnostic and therapeutic algorithm for bilateral diaphragm weakness (outside of the intensive care setting). VC, vital capacity; MIP, maximal inspiratory pressure; TF, thickening fraction of the diaphragm; NPPV, non-invasive positive pressure ventilation; PaCO_2_, arterial partial pressure of carbon dioxide; SpO_2_, peripheral oxygen saturation; Pdi,tw, twitch transdiaphragmatic pressure.

Figure 1 and Figure 2 summarise the suggested diagnostic and therapeutic algorithms for uni- and bi-lateral diaphragm dysfunction.

## 8. Conclusions

Diaphragm weakness, whatever its cause, is often associated with negative clinical repercussions. A thorough evaluation is required to identify its origin and properly manage its effects on symptoms, sleep homeostasis and exercise capacity. The increasing availability of ultrasound has provided a simple and effective means of routinely evaluating diaphragm function that should help clinicians orientate the patient towards adequate treatment, if required. Referral to a center with experience in this disease and access to diaphragm ultrasound, phrenic stimulation or pacing and surgical expertise in diaphragm plication should be considered, where applicable.

## Figures and Tables

**Table 1:** Common alternative causes of an elevated hemidiaphragm image on a chest X-ray.

“False” Hemidiaphragm Paralysis	Extra-Diaphragmatic Disease
Bochdalek hernia	Pulmonary or mediastinal mass
Morgagni hernia	Subphrenic abscess
Traumatic rupture	Ascites
Hiatal hernia	Pulmonary embolism, atelectasis
Lipomas	Asymmetrical emphysema
Eventration	
Lung resection	

**Table 2:** Principal causes of unilateral diaphragm weakness (partial or complete loss of contractility).

Compressive or Infiltrative Processes	Inflammatory Disease
Mediastinal or pulmonary malignancy	Shingles
Pathological lymph nodes	Parsonage-Turner syndrome
Goiter	Mononeuritis
Cervical arthrosis and spondylosis	Chronic inflammatory demyelinating polyneuropathy
	Post-viral
**Traumatic lesions**	
Heart surgery	**Central neurological disease**
Cervical/neck surgery	Stroke
Lung/heart/liver transplant	Rhizotomy
Chiropractic manipulation	Multiple sclerosis
Central venous cannulation	
Nervous blockade	**Idiopathic**

**Table 3:** Principal causes of bilateral diaphragm weakness (partial or complete loss of contractility).

Neurological Disease	Myopathy
Medullary transection	Muscular dystrophies
Multiple sclerosis	Dysthyroidism
Amyotrophic lateral sclerosis	Malnutrition
Severe cervical spondylolysis	Amyloidosis
Poliomyelitis	Post-viral
Guillain-Barré syndrome	Critical illness/ventilator-induced diaphragm dysfunction
Chronic inflammatory demyelinating polyneuropathy	Corticosteroid use
	Disuse atrophy/inactivity
**Idiopathic**	
	**Connective-tissue diseases**
	Systematic lupus erythematosus/shrinking lung syndrome
	Dermatomyositis
	Mixed connective-tissue disease
